# A New Role for an Old Antimicrobial: Lysozyme c-1 Can Function to Protect Malaria Parasites in *Anopheles* Mosquitoes

**DOI:** 10.1371/journal.pone.0019649

**Published:** 2011-05-06

**Authors:** Mayur K. Kajla, Lei Shi, Bin Li, Shirley Luckhart, Jianyong Li, Susan M. Paskewitz

**Affiliations:** 1 Department of Entomology, University of Wisconsin, Madison, Wisconsin, United States of America; 2 Section of Allergy, Pulmonary and Critical Care, Department of Medicine, University of Wisconsin School of Medicine and Public Health, Madison, Wisconsin, United States of America; 3 Section of Microbiology, College of Biological Sciences, University of California Davis, Davis, California, United States of America; 4 Department of Medical Microbiology and Immunology, University of California Davis, School of Medicine, Davis, California, United States of America; 5 Department of Biochemistry, Virginia Tech, Blacksburg, Virginia, United States of America; Indian Institute of Science, India

## Abstract

**Background:**

*Plasmodium* requires an obligatory life stage in its mosquito host. The parasites encounter a number of insults while journeying through this host and have developed mechanisms to avoid host defenses. Lysozymes are a family of important antimicrobial immune effectors produced by mosquitoes in response to microbial challenge.

**Methodology/Principal Findings:**

A mosquito lysozyme was identified as a protective agonist for *Plasmodium*. Immunohistochemical analyses demonstrated that *Anopheles gambiae* lysozyme c-1 binds to oocysts of *Plasmodium berghei* and *Plasmodium falciparum* at 2 and 5 days after infection. Similar results were observed with *Anopheles stephensi* and *P. falciparum*, suggesting wide occurrence of this phenomenon across parasite and vector species. Lysozyme c-1 did not bind to cultured ookinetes nor did recombinant lysozyme c-1 affect ookinete viability. dsRNA-mediated silencing of *LYSC-1* in *Anopheles gambiae* significantly reduced the intensity and the prevalence of *Plasmodium berghei* infection. We conclude that this host antibacterial protein directly interacts with and facilitates development of *Plasmodium* oocysts within the mosquito.

**Conclusions/Significance:**

This work identifies mosquito lysozyme c-1 as a positive mediator of *Plasmodium* development as its reduction reduces parasite load in the mosquito host. These findings improve our understanding of parasite development and provide a novel target to interrupt parasite transmission to human hosts.

## Introduction

*Plasmodium* parasites, the etiologic agents of malaria, display a complex life cycle with multiple forms occurring in the vertebrate and invertebrate hosts. In the mosquito, parasites undergo sexual reproduction and develop through several stages within the gut before transforming into mobile ookinetes that cross the gut epithelium. The ookinetes reach the basal lamina surrounding the gut and then transform into oocysts. The oocyst undergoes multiple asexual divisions, resulting in thousands of haploid sporozoites, which eventually are released from the oocyst into the circulation. Sporozoites invade the salivary glands after crossing through the cells in transit to the central ducts of the gland.

During the insect phase of the *Plasmodium* life cycle, parasites must survive for longer than a week in the body of the insect. Mosquitoes have the ability to mount a strong defense that kills many parasites, as illustrated by the dramatic increase in number of parasites when certain antagonistic genes of the mosquito are silenced through RNA interference [1]–[3]. By contrast, *Plasmodium* interacts with other mosquito proteins in ways that promote parasite development, since silencing of these genes results in a reduction in the number of surviving parasites [4]–[5]. Some of these positive factors appear to play roles in the formation of the oocyst [5]–[6] or in ookinete penetration of the cells [3] while the function of others is not defined [2]. Both types of regulators of parasite development offer new targets for malaria control, since transmission could be blocked by promoting negative regulators or by interfering with positive interactions.

Lysozymes (EC 3.2.1.17) are antibacterial proteins defined by their ability to hydrolyze β-1, 4-glycosidic linkage between *N*-acetylmuramic acid and *N*-acetylglucosamine of peptidoglycan in the cell wall of bacteria. This generally limits direct growth inhibition to Gram-positive bacteria [7] although there are a growing number of exceptions [8]–[10]. In the animal kingdom three distinct categories of lysozymes have been identified, c-type, g-type and i-type [11]. C-type lysozymes are widely distributed among animals, with isoforms in taxa ranging from mammals to arthropods. In mosquitoes as in other dipteran insects, multiple lysozyme genes exist, only some of which are inducible by microbial challenge [12]–[14]. In *An. gambiae*, the genome encodes eight c-type lysozyme genes [15]. One of these, lysozyme c-1, binds to and can protect an abiotic target (CM-Sephadex beads) from melanization in *An. gambiae* [16] and is induced upon bacterial challenge [15], [17]–[18]. Lysozyme c-1 is a typical antibacterial protein and exhibited muramidase activity against the Gram-positive bacteria *Micrococcus luteus* and *Bacillus subtilis* [19]. Silencing of *LYSC-1* by RNAi resulted in enhanced mortality in the mosquitoes following bacterial challenge [19].

Here, we report the surprising finding that an *Anopheles gambiae* lysozyme acts as a protective agonist for the development of *Plasmodium* oocysts. In the studies presented here, immunohistochemical analyses and gene silencing confirmed that physical interaction of lysozyme c-1 with the parasite surface following the critical period of midgut invasion was associated with parasite persistence. Identification of this mosquito protein as a positive agonist for malaria parasite development – a novel finding for an antibacterial effector protein – provides a new target for interference with the oocyst stage of the parasite life cycle.

## Results

### Lysozyme c-1 binds to oocysts of *P. berghei* and *P. falciparum*

In previous studies we used CM-Sephadex beads as a tool to study melanization of foreign targets in *An. gambiae* [16], [20]–[21]. We discovered that the L3-5 strain of *An. gambiae* usually melanized both malaria parasites and CM-Sephadex beads, while a susceptible strain (4a rr) did not. Beads were protected from melanization upon transfer from 4a rr to L3-5 females [21] suggesting that the protective factor was bound to transferred beads. Lysozyme c-1 was identified in eluates from beads that were incubated in 4a rr mosquitoes and knockdown of the *LYSC-1* gene in the 4a rr strain restored melanization upon transfer to L3-5 [16]. These studies suggested that physical association of lysozyme c-1 with developing malaria parasites might protect them from mosquito defense responses [22].

**Figure 1:**
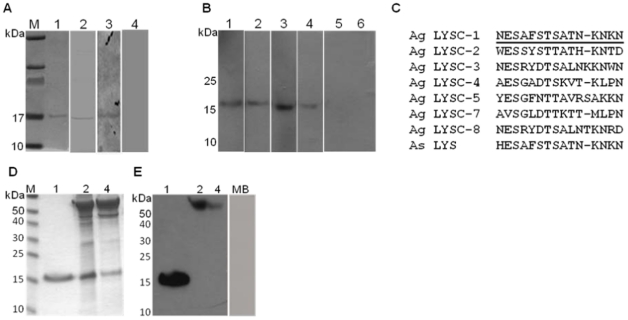
Anti-lysozyme c-1 antibodies 9122 and 9124 specifically recognized lysozyme c-1. (A) Western blot showing cross-reaction of lysozyme c-1 antibodies with salivary glands from *An. gambiae* G3 (lane 1 with 9122; lane 2 with 9124), with salivary glands from *An. stephensi* (lane 3 with 9122) and no cross-reaction of salivary gland extracts with pre-immune serum (lane 4). (B) Western blot showing cross-reaction of 9122 antibodies (lane 1 and 2) or 9124 (lane 3 and 4) or pre-immune serum (lane 5 and 6) with recombinant lysozyme c-1 purified from *E. coli* (lane 1, 3 and 5) and from conditioned medium of 4a3B cells (lane 2, 4 and 6). (C) Alignment of the *An. gambiae* lysozyme c-1 peptide (underlined) used to generate antibodies with other *An. gambiae* homologues and an orthologue from *An. stephensi*. (D) Coomassie stained gel showing *E. coli* expressed recombinant lysozyme c-1 (lane 1); partially purified recombinant lysozyme c-2 (lane 2) and partially purified recombinant lysozyme c-4 (lane 4). (E) Western blot showing cross-reaction of lysozyme c-1 antibodies (9122) with purified recombinant lysozyme c-1 (lane 1) and absence of cross-reactivity with partially purified lysozymes c-2 and c-4 (lanes 2 and 4) and serum proteins from mouse (lane MB).

To investigate whether lysozyme c-1 (GenBank accession DQ007317) binds to *Plasmodium* parasites in susceptible mosquitoes, we performed immunohistochemical analyses of midgut tissues from mosquitoes infected with *P. berghei* or *P. falciparum*. We verified the specificity of anti-lysozyme c-1 antibodies αLys-c-1 9122 and αLys-c-1 9124 (hereafter designated as 9122 and 9124) against lysozyme c-1 obtained from mosquito salivary glands, conditioned media of *An. gambiae* cell line 4a3B and recombinant lysozyme c-1 produced in *E. coli or* baculovirus. Via Western blotting, we confirmed that these antibodies specifically cross-reacted with a protein approximating the expected molecular weight of 15 kDa (Figure 1A and 1B) in these samples. A pre-immune serum from the same rabbit in which lysozyme c-1 antibodies (9122) were raised did not cross-react to lysozyme c-1 from any of the aforementioned sources (Figure 1A and 1B). Further support for specific binding of these antibodies to lysozyme c-1 was derived from these observations. First, the peptide used for generation of 9122 and 9124 antibodies differed significantly from the sequences of other *An. gambiae* lysozymes at these residues but was nearly identical to the orthologous sequence from *An. stephensi* (Figure 1C, [10]). Second, antibody 9122 did not cross-react with partially purified recombinant lysozymes c-2 and c-4 produced in *E. coli* (Figure 1D and 1E) or with proteins from mouse blood (Figure 1E).

**Figure 2:**
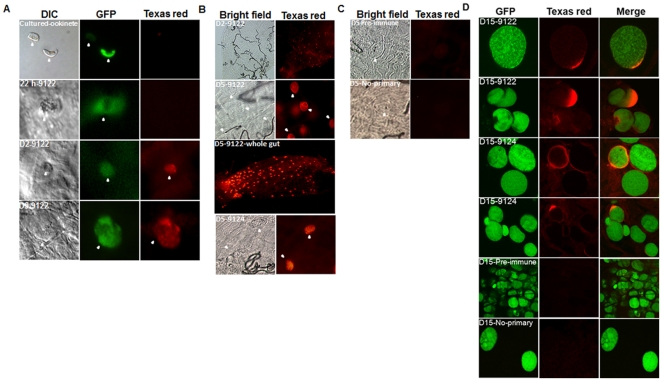
Lysozyme c-1 is associated with *Plasmodium berghei* and *Plasmodium falciparum oocysts*. *Plasmodium berghei* parasites were detected via green fluorescence and anti-lysozyme c-1 antibodies (9122 or 9124 as indicated with white arrows) were detected using Texas-red secondary antibody. All images were collected using confocal microscopy. Representative images of the *P. berghei* and *P. falciparum* parasites (indicated with white arrows) are shown. (A) Lysozyme c-1 was not detected on early stage ookinetes of *P. berghei* (22 h, upper left) but was present on later stages (Days 2 and 5; D2 and D5) of both *P. berghei* (A, lower left) and *P. falciparum* oocysts in *An. gambiae* (B). Pre-immune serum as well as omitting primary antibody showed no staining of parasites (C) indicating a specific labeling by anti-lysozyme antibody 9122 or 9124. Day 5 parasites are shown for control antibodies. (D) Staining of *P. berghei* oocysts with 9122 as well as 9124 antibodies at day 15 post-infection in *An. stephensi*. DIC = differential interference contrast; GFP = Green fluorescent protein; Texas red = Goat anti-rabbit secondary antibodies labeled with Texas-red used to visualize 9122 or 9124 primary antibodies for lysozyme c-1.

We surveyed over 300 parasites from nine separate infections of *P. berghei* in *An. gambiae*, more than 700 parasites from two infections of *P. falciparum* in *An. gambiae*, and 30 parasites from one infection of *P. falciparum* in *An. stephensi* for lysozyme c-1 labeling using anti-lysozyme c-1 antibodies. *In vivo*, some ookinetes showed a variable degree of labeling but 80–90% ookinetes were not labeled at 22–24 h (Figure 2A). Similarly, *in vitro* cultured ookinetes pre-incubated with recombinant lysozyme c-1 did not subsequently cross-react with these antibodies. In contrast, the 9122 and 9124 antibodies bound to nearly all oocysts of *P. berghei* (Figure 2A) and *P. falciparum* (Figure 2B) *in vivo* at 2 days and 5 days post-infection. Additionally about 12% of *P. berghei* oocysts counted from eight infected *An. stephensi* midguts showed lysozyme c-1 staining at day 15 post-infection (Figure 2D). Among the 15 day old oocysts that exhibited this lysozyme c-1 signal, some had lysozyme c-1 around the capsule while others had intense staining concentrated at one edge of the parasite. No lysozyme c-1 staining was detected in controls where pre-immune sera were used as the primary antibody or when primary antibody was omitted (Figure 2C and 2D lower panels) confirming a specific binding to lysozyme c-1 on the parasite surface.

We considered the possibility that the antibody might bind to a vertebrate lysozyme, since the ookinetes first develop in vertebrate blood within the gut. However, the lysozyme c-1 antibody (9122) did not cross-react with any mouse serum proteins (Figure 1F, lane MB) nor did it bind to ookinetes cultured from mouse blood (results were similar to absence of texas-red signal as presented in Figure 2, upper left panel).

### Lysozyme c-1 is necessary for *P. berghei* oocyst development in *An. gambiae*

**Figure 3:**
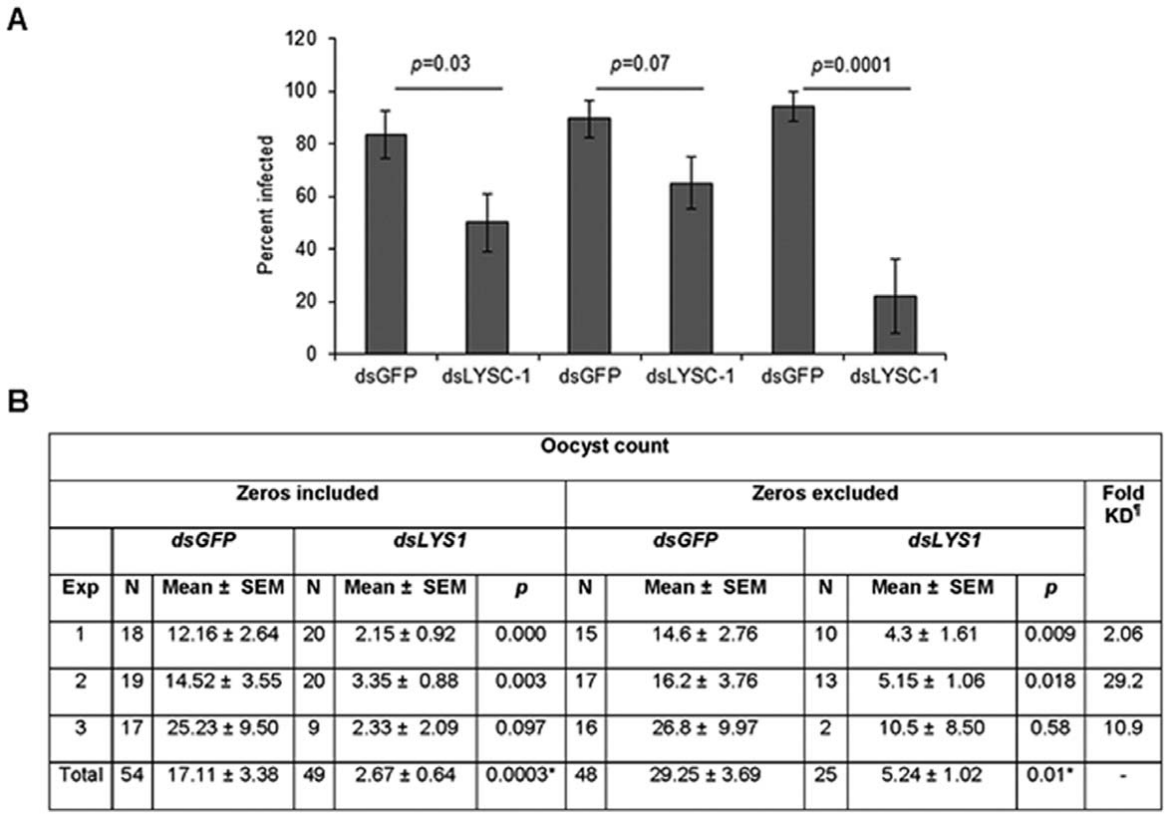
Lysozyme c-1 knockdown (KD) significantly reduced prevalence and intensity of *P. berghei* infections. The results of three independent replicate experiments for the *An. gambiae* G3 strain are shown. (A) Shows the significantly decreased prevalence of infection in the *LYSC-1* KD mosquitoes. Prevalence was defined as the percentage of mosquitoes that had at least one oocyst per mosquito midgut. (B) Oocyst count data from the three independent replicate experiments including zeros or excluding zeros (mosquitoes that had no infection) is presented. A two-way ANOVA was conducted on individual experiments and *p*-values are presented along with mean ± SE of oocysts counted in each experiment. The *p*-value for the totals row indicates the significance of the test when the effect of experiment was included. Parasite numbers were significantly lower in the *LYSC-1* KD mosquitoes as compared to control *GFP* group. ^¶^Indicates the fold reduction in the expression *LYSC-1* as compared to control *GFP* groups as determined via densitometry analysis (see Materials and Methods).

**Figure 4:**
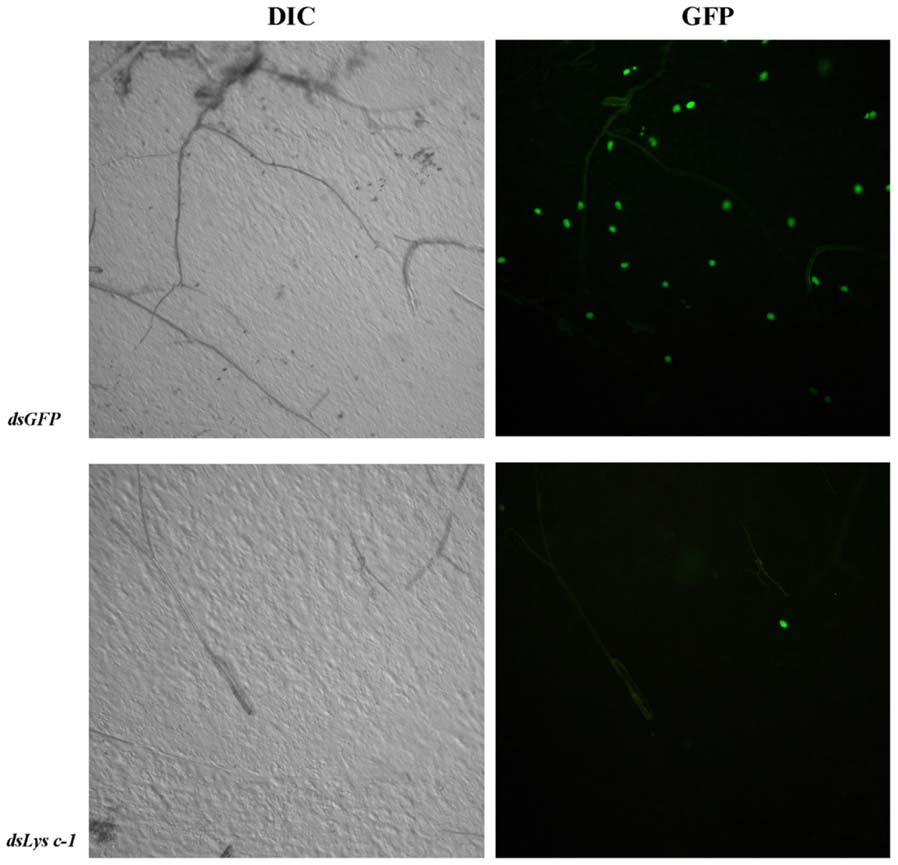
Lysozyme c-1 knockdown did not reduce *P. berghei* oocyst development via increased melanization. Comparison of midguts infected with a GFP-expressing strain of *P. berghei* four days after the mosquitoes were injected with either *dsGFP* or *dsLYSC-1*. At 3 days post-infection viable parasites were dramatically reduced (GFP) but parasites did not become melanized (DIC).

Injection of dsRNA into the thorax of female *An. gambiae* G3 mosquitoes significantly reduced the *in vivo* expression of *LYSC-1* in all three replicate experiments (2–30 fold; Figure 3). Four days after dsRNA injection, *An. gambiae* G3 mosquitoes were allowed to feed on mice infected with GFP-expressing *P. berghei*. Infection levels were scored by counting the number of oocysts per midgut at 3 days post-infection. In three independent experiments, knockdown of *LYSC-1* significantly reduced prevalence (Figure 3A) and intensity (Figure 3B) of *P. berghei* infections. The reduction in prevalence of infection in *LYSC-1 KD* mosquitoes ranged from 33–72% relative to *GFP* controls. Further, *LYSC-1* KD caused a 6.4 fold reduction (*p* = 0.0003) in the average number of oocysts counted from three separate experiments as compared to *GFP* controls. There was no effect on the statistical significance when we performed analysis on data including all parasite fed mosquitoes (including zeros) or data omitting uninfected mosquitoes (excluding zeros) (Figure 3). Because lysozyme c-1 can protect Sephadex beads from melanization [11] we also looked for the occurrence of melanized parasites. None were identified in either *GFP* or *LYSC-1* KD mosquitoes (Figure 4).

In order to determine whether lysozyme c-1 mediated midgut invasion or very early oocyst development, we quantified prevalence and intensity of *P. berghei* infection in *An. gambiae* at 24 h post-infection, a time point that corresponds with early midgut invasion and initial oocyst formation. Neither the prevalence of infection nor the number of ookinetes per midgut were statistically different between *LYSC-1* and *GFP* KD groups [average number of ookinetes in *dsLYSC-1* injected (n = 17) = 110 and *dsGFP* injected mosquitoes (n = 19) = 127, T-test *p* = 0.59].

Finally we examined whether lysozyme c-1 would enhance the viability of cultured ookinetes. A time course of incubation of cultured ookinetes with *in vitro* produced lysozyme c-1 showed no effect on the viability of the ookinetes over a 24 h period. We found a little change in the number of dead parasites between 2 and 24 h. In the control sample (ookinetes incubated with BSA) 6.3% ookinetes were dead by 24 h compared with 5.1% by 2 h. In the lysozyme c-1 treated sample 3.85% ookinetes were dead by 24 h as compared with 3.80 by 2 h.

### Neither lysozyme c-1 or its muramidase activity were detected in the midgut tissues

**Figure 5:**
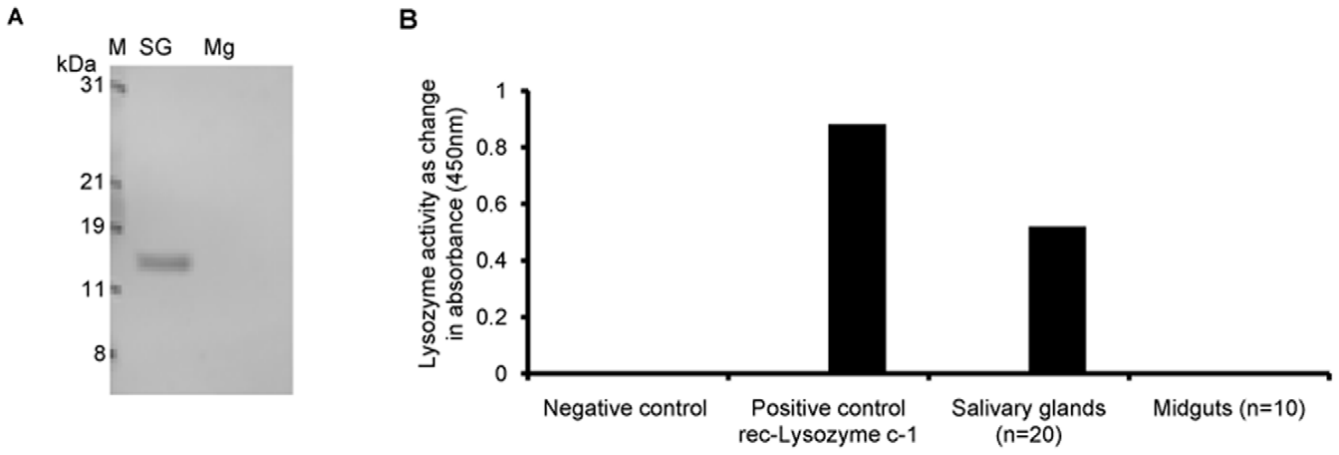
Lysozyme c-1 and its muramidase activity were non-detectable in *An. gambiae* midguts. (A) A representative Western blot showing an absence of protein cross-reactivity with 9122 antibodies in the midguts as compared to salivary glands. (M = Protein marker). (B) Turbidometric assay showing absorbance values of different samples subtracted from control (which contained only buffer). Muramidase activity was non-detectable in the midgut tissues (n = 10) as compared to positive control (*E.coli* produced recombinant lysozyme c-1) as well as salivary glands (n = 10 pairs) 24 h after incubation with *Micrococcus lysodeikticus* cells.

The association of lysozyme c-1 with developing parasites suggested that this protein could be synthesized or localized to the midgut, an observation that would have extended previous identifications of lysozyme c-1 in hemolymph, salivary glands and hemocytes [16], [19], [23]. To investigate this possibility, we examined dissected midguts for lysozyme c-1 protein localization and muramidase activity. On repeated trials we could neither detect a lysozyme c-1 signal on midgut Westerns nor any muramidase activity in midgut extracts (Figure 5). It is possible that elevated protease activity in the midgut extracts might cause a rapid proteolysis of this protein. However, inclusion of protease inhibitors did not improve the results.

### Depletion of *LYSC-1* via gene silencing did not alter the overall number of culturable midgut bacteria in *An. gambiae*

**Figure 6:**
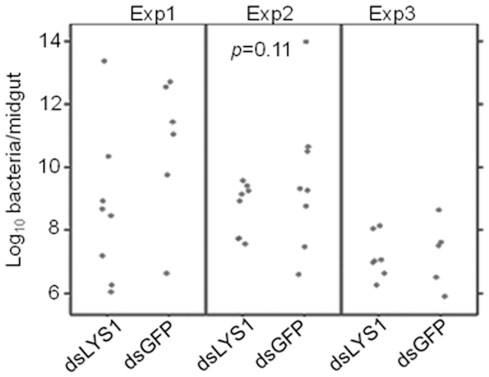
Silencing of *LYSC-1* did not alter the overall number of culturable bacteria in *An. gambiae* midguts. Enumeration of culturable bacteria in the midguts of *dsGFP* and *dsLYSC-1* injected mosquitoes. The presented bacterial count data were derived from individual mosquitoes from three biological replicates. Statistical analysis on the count data was performed using software suite R. A two-way ANOVA analysis on the log-transformed data did not reveal any statistical significance in the total number of bacteria in the *LYSC-1* knockdown mosquitoes as compared to *dsGFP* injected mosquitoes (*p* = 0.11).

Lysozyme c-1 is an antibacterial protein and, as such, we recognized that its silencing could alter the growth of the midgut microbiota that has been shown to affect *Plasmodium* success in infected mosquitoes [18]. To examine this possibility, we tested whether depletion of *LYSC-1* affected midgut bacteria. Silencing of *LYSC-1* did not result in a statistically significant difference in the total numbers of bacteria between control *dsGFP* versus *dsLYSC-1* groups (Figure 6).

## Discussion

The sporogonic development of malaria parasites depends on a complex interaction with their mosquito hosts. Although lysozymes are well known for their antimicrobial activities, our data demonstrated that the *An. gambiae* lysozyme c-1 is a positive regulator of *Plasmodium* parasite development. Knockdown of the *LYSC-1* gene significantly reduced the prevalence of *P. berghei*-infected *An. gambiae* as well as the average number of oocysts per infected midgut. Immunohistochemistry clearly demonstrated that lysozyme c-1 was associated with oocysts of *P. falciparum* and *P. berghei* in both *An. gambiae* and *An. stephensi*. Knockdown of *LYSC-1* in *An. gambiae* mosquitoes did not result in changes in numbers of viable *P. berghei* parasites until 3 days post-infection. That is, similar numbers of fluorescing parasites were seen in control and knockdown mosquitoes at 24 h post-infection. This suggested that formation of ookinetes and invasion of the midgut were similar in treated and control mosquitoes, and that the block occurred after oocyst formation. Based on these observations we conclude that lysozyme c-1 is important to the successful persistence of this parasite stage.

In addition to lysozyme c-1, other positive regulators of parasite development have been identified. After ookinetes localize between the epithelial cells and the midgut basal lamina (BL), the transition to oocysts occurs. The rapidly expanding oocysts stretch the overlying layer of the BL at the hemocoelic surface while a new BL is generated between the oocysts and the epithelial cells [24]. At the same time, mosquito-derived collagen and laminin (types of extracellular matrix proteins), are incorporated into oocyst capsules [2], [23], [25]–[26]. Knockdown of laminin mRNA led to a substantial reduction in the number of successfully developed oocysts [5]. Laminin has been shown to bind to at least five *P. berghei* proteins (P25, P28, SOAP, circumsporozoite and TRAP-related) in yeast two hybrid assays [24], [26]–[27]. Recently Nacer et al. [6] convincingly showed that mosquito-produced laminin indeed becomes part of the parasite capsule during its passage through the gut.

The acquisition of the basal lamina proteins is likely to help protect the developing oocysts from the mosquito immune system and, therefore, may facilitate their prolonged extracellular development in the mosquito body cavity [25]. Could one of these protecting proteins be lysozyme? Vertebrate lysozymes bind to glycosaminoglycans in extracellular matrices [28] and insect basal laminae are negatively charged [29] which could promote interaction with the basic lysozyme c-1. Lysozymes have also been shown to bind and prevent the proteolytic degradation of the elastin component of elastic fibers in the basal lamina, indicating that lysozyme interaction can protect elastic fibers at the sites of injury [30]. Arrighi et al. [5] suggested that the production of new basal lamina around the midgut may be a normal process following blood feeding, a process that has been co-opted by the parasite. Thus, we hypothesize that lysozyme c-1 might associate with components of the midgut BL and become incorporated during formation of the BL-related capsule around the parasite.

Our immunohistochemistry data on the interaction of lysozyme c-1 and malaria oocysts support a direct lysozyme c-1 association with the parasite. The protein may not originate from the midgut cells, since we failed to detect the protein in Western blots and could not detect muramidase activity even after extended incubation periods of midgut extracts. Ahmed et al. [31] also failed to detect muramidase activity in midgut extracts following blood feeding. By contrast we detected lysozyme c-1 in mosquito hemolymph through Western blotting [16], [19] and Ahmed et al. [31] determined that muramidase activity in the hemolymph increased following blood feeding. Castillo et al. [23] also described the occurrence of lysozyme c-1 in hemocytes. These observations suggest that lysozyme c-1 associated with parasites is derived from the hemolymph . In studies of the transport of molecules from the hemolymph across the basal lamina to the intercellular spaces of the midgut epithelium, other researchers have shown that cytochrome-c can make this passage [32]. Cytochrome-c is nearly identical to lysozyme c-1 in both size and charge. Thus, it is entirely possible that lysozyme c-1 can also move in this direction.

Recent work has described the impact of microbial communities inhabiting the mosquito midgut on *Plasmodium* development [18], [33]. We considered the possibility that the antibiotic properties of lysozyme c-1 might alter the microbial community of the gut and thereby contribute to the proliferation and protection of *P. berghei* parasites in *An. gambiae*. However, we did not find a significant change in the numbers of culturable bacteria in control mosquitoes when compared with mosquitoes depleted of *LYSC-1*. This result was not surprising since our previous work demonstrated that purified lysozyme c-1 had little effect on bacteria isolated from midguts [19]. This evidence plus the absence of detectable lysozyme c-1 protein or muramidase activity in the midguts support the hypothesis that the direct association of hemolymph-derived lysozyme c-1 and the oocyst is key to the agonistic interaction. Future studies will investigate the interaction of malaria parasites and lysozyme c-1 in the light of these findings.

## Materials and Methods

### Ethics statement

This study was carried out in strict accordance with the recommendations in the Guide for the Care and Use of Laboratory Animals of the National Institutes of Health. The protocol was approved by the University of Wisconsin-Madison Animal Care and Use Committee (ACUC) under the assurance number A3368-01. All experiments were performed under anesthesia and all efforts were made to minimize suffering.

### Mosquitoes and *Plasmodium* parasites (*P. berghei* and *P. falciparum*)

*Anopheles stephensi* and the G3 strain of *An. gambiae* were used in *P. berghei* feeding experiments and mosquitoes were reared as described previously [34]. Mosquitoes were infected with *P. berghei* (ANKA, GFP-labeled; [35]) as previously described [34] and were maintained at 21°C with 80% humidity to allow parasite development. Transgenic parasites expressing GFP have been widely used as a laboratory model to study mosquito-parasite interactions and have been shown to behave like wild-type parasites [36]. *Anopheles gambiae* (Kisumu) and *An. stephensi* (Indian wild type) were reared at UC Davis, CA, USA according to guidelines published in [35] and were used for infections with *P. falciparum* NF54 strain [37]. Mosquitoes infected with *P. falciparum* were maintained at 26°C with 80% humidity.

GFP-labeled *P. berghei* was passaged in Balb/c female mice. Parasitemia was determined from Giemsa-stained blood smears and exflagellation was determined with phase contrast microscopy. For *P. berghei*, 3–5 day old mosquitoes were fed at parasitemia of 4–5% and exflagellation rate about 10 exflagellation events in 20 microscopic fields. Mosquitoes were dissected in PBS and immunohistochemical analysis was performed on midguts as described below. Mosquito midguts were dissected 22 h (early stage parasites just started to invade midgut epithelium), 2 and 5 days (during early oocyst satges) and 15 days (during mid to late oocyst stages) post infection. The luminal contents were removed in PBS. Morphology of the parasites was examined under the fluorescent microscope. There is a distinguishable morphological difference between these ookinete and ooycyst stages of the parasite. Whereas the ookinetes are elongated or retort in shape, the oocysts are oval/round. The numbers of ookinetes/oocysts were counted using a fluorescent microscope (Leica) based on their morphological difference.

*In vitro* culturing of *P. berghei* ookinetes was performed according to protocol described by Winger et al. [38]. For the assessment of lysozyme c-1 effect on ookinete viability, the cultured ookinetes were incubated with *in vitro* produced lysozyme c-1 (100 µg/ml) or similar amounts of bovine serum albumin as control for a period of 2–24 h. Ookinete viability was determined via fluorescence microscopy. Whereas live ookinetes exhibited green fluorescence, dead parasites did not fluoresce green but were visible under phase contrast optics.

### Synthesis of peptides for antibody production and Western blotting

To produce antibodies (9122 and 9124) for lysozyme c-1, peptide sequences for the eight *An. gambiae* lysozymes were aligned, and a fragment of 15 amino acids that was largely unique to lysozyme c-1 was identified (Figure 1D). This peptide was synthesized, conjugated to MAP (a proprietary carrier from Invitrogen, Grand Island, New York), and used for immunization of rabbits by Invitrogen. Rabbits were immunized with 0.5 mg synthetic peptide in adjuvant and then boosted with the same amount of peptide at weeks 2, 7 and 8. Sera were harvested 10 weeks after initiation of the protocol. Pre-immune serum was collected before the start of immunization. Western blotting was performed either as described previously [19] or with modifications. These modifications included use of primary antibodies, 9122 or 9124, were used at 1∶1,000 dilution and detection of these antibodies with 1∶50,000 dilution of peroxidase- labeled goat-anti-rabbit secondary antibodies using chemiluminiscence substrate (Perkin Elmer, USA) as per supplied instructions along with the kit. Recombinant lysozyme c-1 produced in *E. coli* had a slightly higher molecular weight as it retains a few additional amino acids from expression vector (Figure 1B). (Figure 1D). To assess the specificity of these antibodies, we also tested them against the mouse serum proteins via Western blotting (Figure 1E, lane MB). The negative cross-reaction to *in vitro* cultured ookinetes was also accessed via IHC. The results of these analyses were similar to the lack of red florescence on the ookinetes as presented in Figure 2A, upper left panel.

### Immunohistochemistry (IHC) on dissected mosquito midguts

Immunostaining was performed according to the protocol described by Han *et al*., 2000 [39] with some modifications. Briefly, midguts from parasite-infected mosquitoes at days 2, 5 and 15 post infection, were dissected in phosphate-buffered saline (PBS; 130 mM NaCl, 7 mM Na_2_HPO_4_, 3 mM NaH_2_PO_4_ H_2_O pH 7.2). The tissue was fixed in 4% paraformaldehyde, 100 mM PIPES buffer pH 7.4, 2 mM MgSO_4_ and 1 mM EGTA for 10 min at room temperature. The midguts were rinsed in PBS, then permeabilized in PBT (PBS containing 0.1% Triton X-100) for 5 min. The tissues were blocked in 1% bovine serum albumin in PBT for 2 h at room temperature and incubated with primary antibodies (9122 and 9124) at 1∶200 dilutions in 1% BSA in PBT overnight at 4°C. The tissues were washed with PBT three times 10 min each, and then incubated with Texas-red labeled goat anti-rabbit IgG (Kirkegaard and Perry Labs, Maryland, USA) at 1∶100 dilution in 1% BSA prepared in PBT for 2 h at room temperature. The stained tissues were washed three times in PBT, 10 min each and analyzed using confocal microscopy (Bio-Rad Radiance 2100 MP Rainbow confocal imaging system; Keck Laboratory for Biological Imaging, University of Wisconsin) for GFP (for *P. berghei*) and Texas-red signals.

### dsRNA mediated silencing of *LYSC-1* in the *An. gambiae*

An *in vitro* transcription template was produced using a one-step PCR protocol as described previously [19]. The primers used to prepare the templates for *An. gambiae LYSC-1* and exogenous *GFP* contained T7 sequences on the 5′ ends of the forward and reverse primers (Fwd 5′-TAATACGACTCACTATAGGGATGAAAGTGTTTTCCACAGTTTTG -3′, Rev 5′- TAATACGACTCACTATAGGGAAAAACAGGAGCTAACATTCGG -3′). These primers were used to amplify a product from mosquito cDNA. The *GFP* sequence (Fwd: 5′-TAATACGACTCACTATAGGGCGTGATCAAGCCCGACA-3′, Rev: 5′-TAATACGACTCACTATAGGGCTTCGGCGTGCTC-3′) was used to amplify a product from *phMGFP* vector (Promega, Madison, Wisconsin). The choice of GFP as the control is unrelated to its use as a transgenic marker for the parasite. Injection of dsGFP does not silence parasite GFP probably because it is introduced well before the introduction of the parasite. In these experiments, use of the exogenous, unrelated GFP dsRNA served two purposes 1) as an injection control to assess the impact of breaching the exoskeleton of the host on processes that might affect parasite development, and 2) to assess whether injection of nucleic acids affected process that might affect development of the parasite. The PCR products were purified using a QIAquick gel extraction kit (QIAGEN Sciences, Maryland, USA) and 1–2 µg of the products were used as templates for transcription. The MEGAscript™ RNAi kit (Ambion, Austin, Texas) was used for transcription and the production of dsRNA following the instruction manual. The dsRNA concentration was measured using micro-spectrophotometry (Nanodrop NT1000, Thermo Fisher Scientific, Waltham, MA). dsRNA preparations were checked for integrity on an agarose gels stained with ethidium bromide. The dsRNA preparations were stored at −80°C until used.

For introduction of dsRNA for *LYSC-1* or control *GFP*, newly eclosed female mosquitoes (less than 24 h after eclosion) were cold anesthetized and injected in the thorax using calibrated glass needles under a dissecting microscope. The needles for injections were produced using glass capillary tubes (Fisher Scientific, USA) pulled with Narishige glass puller (Narishige International USA, Inc., NY, USA). To assess the optimal concentration of dsRNA needed to achieve maximal knockdown of *LYSC-1*, we carried out pilot experiments with dsRNA concentration ranging from 0.6–1.4 µg and determined that persistent and efficient knockdowns were achieved with 1.4 µg of dsRNA in 0.1 µl injected volume. The efficiency of the knockdown was examined using semiquantitative RT-PCR at various time pointsfrom 24 h to 7 days after injection of dsRNA and equally efficient KD of LYSC-1 was detected during these times. We also tested the effect of *LYSC-1* KD on other related lysozymes and did not detect altered expression for any of these genes (data not shown).

Mosquitoes were infected with *P. berghei* parasites four days post-injection of dsRNA-a standard procedure described in literature [1]. This time period allows females to recover from the injury caused by the injections. Three independent replicate experiments were performed. For each experiment, *dsGFP* and *dsLYSC-1* mosquitoes were fed on the same infected mouse for the assessment of the effect of *LYSC-1* silencing on the intensity and prevalence of infections. Three days post infection the GFP expressing oocysts were counted from both groups under a fluorescent microscope. A two way ANOVA test was performed on the count data from both infected or uninfected mosquitoes (with or without zeros) to determine the significance of *LYSC-1* knockdowns as compared to control *GFP* groups (Figure 3). ANOVA did not reveal a significant inter-experimental variation among the three replicate experiments for zero-included and zero-excluded data (*p*>2.0). All analysis were conducted using SAS®v9.3 (SAS Institute, Cary, NC).

### Semiquantitative RT-PCR

Semiquantitative RT-PCR was performed to assess relative transcript abundance in adult female mosquitoes following RNAi. Total RNA was isolated from five *An. gambiae* mosquitoes from respective treatments using MasterPure™ Complete DNA and RNA Purification Kit (Epicentre Biotechnologies, Madison, WI, USA) according to the supplied instructions. Total RNA was treated with RQ1 DNAse (Promega, Madison, WI) at a concentration of 1 unit/µg RNA preparation to remove genomic DNA. RNA concentration was measured with micro-spectrophotometry (Nanodrop NT1000, Thermo Fisher Scientific, Waltham, MA). Five hundred nanograms of total RNA were used to synthesize cDNA using a High-Capacity cDNA Archive Kit. The resulting cDNA was stored at −20°C until use. Primers based on *An. gambiae* cDNA sequences for *LYSC-1* (GenBank: accession no. DQ007317) and *RPS7* [40] were designed using Oligoperfect software (Invitrogen). The S7 ribosomal gene was used to normalize the template taken for PCR. Final concentrations of reagents used for RT-PCR were 1× buffer, 200 µM dNTPs, 0.5 µM each primer, 0.2 µl (4 units) Advantage Taq polymerase (Clontech Laboratories Inc., CA, USA). PCR cycle conditions were: an initial denaturation at 95°C for 3 min; then repeated cycles of 95°C for 30 s, 60°C for 30 s, 72°C for 30 s; and a final extension step at 72°C for 10 min. For *LYSC-1*, 35 cycles were used while 25 cycles were used for *S7*. PCR products were separated on 1% agarose gels and stained with ethidium bromide. The gels were exposed to UV-transillumination and images were captured on a gel-imaging system. Signal intensities of RT-PCR bands in the KD experiments were quantified by densitometeric analysis using the Quantity One Software (version 4.6.8) from Bio-Rad Laboratories (Bio-Rad, Hercules, CA). Fold change in expression was calculated by dividing the ratios of *GFP/S7* control with *LYSC-1/S7* KD signals (Figure 3B).

### Lysozyme c-1 activity assays

Muramidase activity assays with purified lysozyme c-1 or midgut extracts were performed as described in [19]. Briefly, ten midguts were homogenized in 20 mM HEPES, pH 7.5. The gut extract was incubated at room temperature on a rotator shaker with 0.5 mg/ml *Micrococcus lysodeikticus* cells prepared in same buffer for 24 h. Muramidase activity was recorded as reduction in the absorbance measured at 450 nm and compared to positive control (purified lysozyme c-1) or with negative control (20 mM HEPES, pH 7.5 buffer).

### Enumeration of bacteria in midguts of *LYSC-1* silenced *An. gambiae* mosquitoes

Silencing of *LYSC-1* was carried out as described above. For these experiments, mosquitoes from *dsLYSC-1* (n = 23) or *dsGFP* (n = 19) injected groups were harvested after four days post injection. Individual mosquito midguts were dissected under sterile conditions as described in [28]. Midgut extracts were serially diluted in 0.9% NaCl and plated on tryptic soy agar (TSA) plates. Bacterial colony forming units (CFUs) in the range of 30–300/plate were counted after 48 h incubation at 28°C. Three independent experiments were performed and CFUs from individual mosquitoes were averaged across the replicates. Statistical analysis on the count data was performed using software suite R. We performed two-way ANOVA on both raw and log-transformed count data. It was necessary to transform the data to obtain adequate normality as we found a great variation in the number of culturable bacterial CFUs from individual mosquitoes within one experimental replicate as well as among three experiments that skewed the normality. A two-way ANOVA analysis on the log-transformed data did not reveal any statistically significant difference among the total number of bacteria between control *dsGFP* and *dsLYSC-1* injected mosquitoes (*p* = 0.11).

### Sources and expression of lysozymes c-1, c-2 and c-4

Lysozyme c-1 was purified either from conditioned media of 4a3B cells as described in [19] or was cloned into a proprietary cloning vector pVP68K (a gift from Dave Aceti, Center for Structural Genomics, UW Madison) and expressed *in vitro* in *E. coli* BL21(DE3) cells according to standard procedures. Lysozyme c-1 is produced as a fusion protein along with histidine and maltose binding protein (MBP) tags. The lysozyme c-1- MBP-His_8_ –fusion protein was purified via amylose affinity columns (New England Biolabs, MA, USA). Purified lysozyme c-1- MBP-His_8_ –fusion was cleaved with 3C protease (Novagen, USA) to remove MBP (maltose binding protein). The cleavage reaction resulted in the precipitation of lysozyme c-1. The precipitated lysozyme c-1 was then denatured in 4M GdHCl and refolded in a buffer containing HEPES-50 mM, NaCl-250 mM, Glycerol-10%, GSH-30 mM, GSSG-5 mM, final pH adjusted to 8.0. The refolded and enzymatically active lysozyme c-1 was further purified to homogeneity via ion exchange chromatography on CM-Sephadex C-25 beads (Figure 1D). Recombinant lysozyme c-2 and c-4 were generated and partially purified on amylose resin using the same bacterial expression system.
